# Spontaneous rupture of a parastomal hernia with evisceration of small bowel: a case report

**DOI:** 10.1186/s12893-019-0509-5

**Published:** 2019-04-25

**Authors:** Oshan Basnayake, Umesh Jayarajah, Jayan Jayasinghe, Pradeep Kumara Wijerathne, Dharmabandhu Nandadeva Samarasekera

**Affiliations:** 1 Professorial Surgical Unit, National Hospital of Sri Lanka, Colombo, Sri Lanka; 2 Department of Surgery, Faculty of Medicine, University of Colombo, Kynsey Road, Colombo 8, Western Province Sri Lanka

**Keywords:** Parastomal hernia, Spontaneous rupture, Evisceration, Sigmoid loop colostomy, Case report

## Abstract

**Background:**

Long standing ostomy related complications such as parastomal hernia and stoma prolapse may be at a higher risk of developing spontaneous rupture and evisceration, especially in patients suffering from chronic cough. Such patients may need early refashioning of the stoma to prevent this serious complication. Parastomal evisceration is a very rare complication of stomas and to date, only few cases have been reported in the literature.

**Case presentation:**

A 51 year old patient with chronic obstructive pulmonary disease (COPD) and extensive hidradenitis suppurativa of the perineum underwent a temporary defunctioning loop sigmoid colostomy and subsequent perineal skin excision and skin grafting. The ostomy was complicated by a parastomal hernia and stoma prolapse 6 weeks post operatively. Five months later he developed spontaneous rupture of parastomal hernia and evisceration of small bowel. Urgent surgery was done and reduction of small bowel loops and re-siting of the sigmoid colostomy was done.

**Discussion and conclusions:**

Parastomal evisceration is an extremely rare life threatening stoma-related complication which requires urgent treatment.

## Background

The creation of a permanent or temporary stoma is associated with varying complication rates ranging from 21 to 70% [1]. Common complications include ischaemia, skin irritation, retraction, prolapse and parastomal hernia and these are associated with poor quality of life and furthermore, can rarely present as emergencies [2].

Parastomal hernia occurs through an acquired defect of the abdominal wall due to a surgical incision which allows protrusion of abdominal viscera and the incidence differs with the type of intestinal stoma. The reported occurrence of parastomal hernia with loop colostomy is between 0–30.8% [3]. Similar to other abdominal hernias, patients can have complications such as irreducibility, obstruction and strangulation.

Parastomal evisceration is an extremely rare complication with only few cases reported in the literature.

## Case presentation

A 51 year old patient with chronic obstructive pulmonary disease (COPD) due to long term smoking and extensive hidradenitis suppurativa of the perineum underwent a temporary defunctioning loop sigmoid colostomy and subsequent extensive perineal skin excision and skin grafting.

**Fig. 1:**
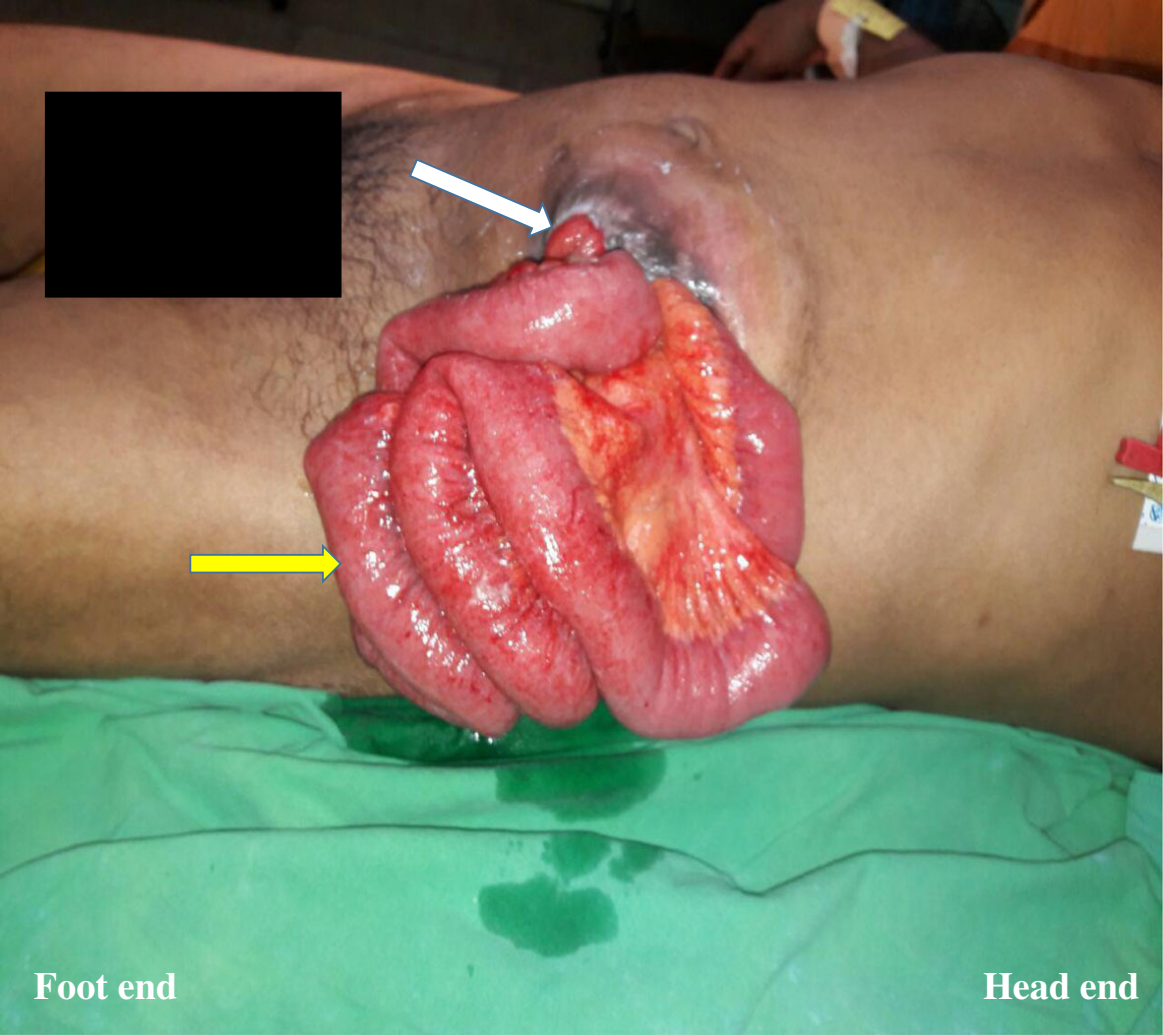
White arrow shows the site of loop sigmoid colostomy and yellow arrow shows the eviscerated small bowel loops

The ostomy was complicated by a parastomal hernia and stoma prolapse 6 weeks post operatively. Conservative management was opted as the stoma was temporary and was functioning well. Five months later, while he was in hospital care for further excision of perianal skin and skin grafting, he developed acute onset pain at the stoma site with rupture of parastomal hernia and evisceration of small bowel loops (Fig. 1).There was no evidence of strangulation or intestinal obstruction. Urgent surgery was done and reduction of small bowel loops and re-siting of the sigmoid colostomy was done. Post-operatively he had exacerbation of COPD which required intensive care management with positive pressure ventilation for 2 days. Thereafter, his recovery was uneventful.

## Discussion and conclusions

Parastomal evisceration is a very rare complication of stomas and to date, only **ten cases have** been reported worldwide. Most of the previously published cases were associated with ileostomies [4–6] and four were reported in association with colostomies [7–10].The majority of the cases were associated with stoma prolapse or parastomal hernia and one within the immediate post-operative period [10].

In our patient, long term parastomal hernia and stoma prolapse may have caused ischaemia and weakening of the underlying abdominal wall and the overlying skin resulting in parastomal evisceration of small bowel. Furthermore, the increased abdominal pressure due to chronic cough(i.e. due to COPD) may have also contributed. Lolis et al. [7] reported a patient with similar contributory factors in a patient with a parastomal hernia and a chronic colostomy prolapse.

Parastomal evisceration is an extremely rare life threatening stoma-related complication which requires urgent treatment. Patients with COPD and long standing ostomy related complications such as parastomal hernia and stoma prolapse may be at a higher risk of developing this complication. Such patients may need early refashioning of the stoma to prevent this serious complication.

## Abbreviations

COPD: chronic obstructive pulmonary disease
